# Decision making improves sperm chemotaxis in the presence of noise

**DOI:** 10.1371/journal.pcbi.1006109

**Published:** 2018-04-19

**Authors:** Justus A. Kromer, Steffen Märcker, Steffen Lange, Christel Baier, Benjamin M. Friedrich

**Affiliations:** 1 cfaed, TU Dresden, Dresden, Sachsen, Germany; 2 Faculty of Computer Science, TU Dresden, Dresden, Sachsen, Germany; Worcester Polytechnic Institute, UNITED STATES

## Abstract

To navigate their surroundings, cells rely on sensory input that is corrupted by noise. In cells performing chemotaxis, such noise arises from the stochastic binding of signalling molecules at low chemoattractant concentrations. We reveal a fundamental relationship between the speed of chemotactic steering and the strength of directional fluctuations that result from the amplification of noise in a chemical input signal. This relation implies a trade-off between steering that is slow and reliable, and steering that is fast but less reliable. We show that dynamic switching between these two modes of steering can substantially increase the probability to find a target, such as an egg to be found by sperm cells. This decision making confers no advantage in the absence of noise, but is beneficial when chemical signals are detectable, yet characterized by low signal-to-noise ratios. The latter applies at intermediate distances from a target, where signalling molecules are diluted, thus defining a ‘noise zone’ that cells have to cross. Our results explain decision making observed in recent experiments on sea urchin sperm chemotaxis. More generally, our theory demonstrates how decision making enables chemotactic agents to cope with high levels of noise in gradient sensing by dynamically adjusting the persistence length of a biased random walk.

## Author summary

Many cells can navigate upwards a concentration gradient of signalling molecules, a process called chemotaxis. Chemotaxis is used e.g. by sperm cells to find the egg. To measure and compare concentrations, cells count stochastic binding events of signalling molecules that diffuse to cellular receptors. Efficient chemotaxis strategies must be adapted to this molecular shot noise of concentration measurements. We reveal a fundamental relationship between the speed of chemotactic steering and the strength of directional fluctuations that result from the amplification of noise. This implies a trade-off between steering fast and steering reliable. Inspired by recent experiments on chemotaxis of sperm cells of marine invertebrates, we develop a theory that allows to efficiently compute optimal chemotaxis strategies. We show that dynamic switching between either fast or reliable steering can substantially increase the probability for a sperm cell to find the egg. Furthermore, the optimal strategy requires only minimal computational capacities of the chemotactic agent, a key constraint for biological cells. More generally, our work demonstrates a benefit of decision making for chemotaxis in the presence of noise, which could inspire optimal control designs for artificial microswimmers.

## Introduction

Motile cells successfully navigate in external concentration fields of signalling molecules by steering in the direction of local concentration gradients—a process termed chemotaxis. Chemotaxis represents a biological implementation of a gradient-ascent algorithm and is used by bacteria to find food [1], immune cells to locate infection sites [2], and sperm cells to follow gradients of chemical cues to find the egg [3, 4]. The very task of reliably measuring a local concentration gradient with sufficient accuracy is non-trivial at dilute concentrations, since molecular shot noise corrupts concentration measurements [5–8]. To measure a concentration, cells must count individual binding events of signalling molecules, which represents a stochastic Poisson process.

Pioneering work on this topic studied the chemotaxis of mobile agents with advanced information processing skills [9], or even a capacity to compute spatial maps of maximum likelihood of target position [10]. It is an open question how biological cells with limited information processing capability deal with noise during their chemotaxis [11–13].

Here, we present a theory of optimal chemotaxis strategies in the presence of noise, using the framework of Markov decision processes (MDP). First, we compute optimal strategies for the idealized case where cells have perfect knowledge of their distance to the target and their swimming direction. From this, we derive a heuristic for the realistic case where cells only possess a noisy estimate of their positional state.

**Fig 1:**
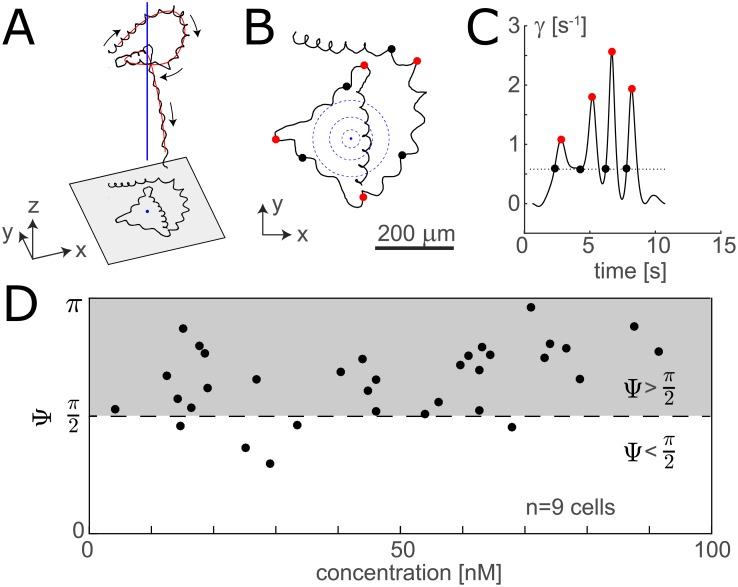
Decision making in chemotaxis of sea urchin sperm. (A) Helical swimming path of a sea urchin sperm cell (black) with helix centreline (red), while navigating in a concentration field of the chemoattractant resact [16]. The concentration field is cylindrically symmetric with symmetry axis parallel to the *z*-axis (indicated in blue). (B) Projection of the same swimming path on the *xy*-plane. Dots mark the beginning (black) and peak (red) of ‘high-gain’ steering phases (or off-responses [16]). The concentration field is indicated by blue circles. (C) From the swimming path and the local gradient direction, we can determine a time-dependent rate *γ*(*t*) of helix bending towards the gradient [16]. The beginning of a ‘high-gain’ steering phase is defined as the level-crossing of *γ*(*t*) above its median as is indicated by black dots. Peaks of *γ*(*t*) are indicated by red dots. (D) Scatter plot of the orientation angle Ψ and local concentration *c* at the beginning of ‘high-gain’ steering phases (*n* = 9 cells). ‘High-gain’ steering is predominantly initiated for Ψ > *π*/2 (grey shading).

We apply this general approach to chemotaxis along helical swimming paths, which is employed by sperm cells of marine species. Chemotaxis along helical paths represents one of the three fundamental gradient-sensing strategies of biological cells [4]. This strategy, also known as helical klinotaxis, is based on temporal comparison of a concentration signal traced along the swimming path [14–16]. By swimming along a helical path, i.e. circling around a centreline, these cells receive information about the gradient component perpendicular to their direction of net motion. In sperm cells, a chemotactic signalling system processes this information and dynamically adjusts the shape of flagellar bending waves [17]. This feedback loop enables cells to steer in a directed manner, by bending the direction of their helical paths towards the local gradient, see also Fig 1A. Helical swimming represents a stereotypical form of exploratory behaviour, employed by sperm cells and other microswimmers [18]. This strategy is typical for sperm cells from species with external fertilization [16, 19, 20]. Helical chemotaxis is qualitatively different from ‘run-and-tumble’ chemotaxis along biased random walks, employed e.g. by swimming bacteria. These bacteria measure only the gradient component parallel to their swimming path [1], and not the perpendicular component, and thus lack the information required for directed steering responses.

The model system of sperm chemotaxis is particularly suited to address optimal navigation in the presence of noise: First, sperm cells have a single objective, to find the egg. In species with external fertilization, evolution presumably optimized the probability to find an egg. Second, recent experiments revealed that sea urchin sperm cells dynamically switch between two different steering modes [16], thus providing an instance of decision making at the scale of individual cells. To date, the benefit of this decision making is not known. With our theory, we demonstrate a benefit of decision making in sperm chemotaxis, and show that this benefit is directly related to noise in cellular gradient sensing.

Our work addresses the intermediate case between the well-understood case of perfect chemotaxis in the absence of noise (perfectly reliable steering), and purely random search strategies that operate in the absence of directed signals (no steering) [21–24]. Random search strategies, such as random walks or Lévy walks are relevant far from a target, i.e. outside the spatial range of chemosensation. For such random search problems, ballistic motion maximizes the rate of finding targets if targets are distributed randomly in an unbounded search domain and can be visited only once, whereas Lévy walks maximize this rate when the same target can be revisited [25]. Here, we are interested in navigation in the vicinity of a target, where chemical signals, though noisy, provide information to the navigating cell.

We show that even if the signal-to-noise ratio of gradient-sensing is below one, thus impeding reliable chemotactic steering, situation-specific switching between two steering modes can substantially increase the probability to find a target, such as the egg. This applies in particular at intermediate distances from the target, in a ‘noise zone’, which cells have to cross before they can perform reliable chemotaxis close to the target.

Our theory highlights a fundamental relationship between the speed of this chemotactic re-orientation and the strength of directional fluctuations, which result from the amplification of noise in the chemotactic input signal.

## Results

### Decision making in sperm chemotaxis: Previous experiments

Recent experiments revealed that during their chemotaxis along helical paths, sea urchin sperm cells switch between two distinct steering modes in a situation-specific manner [16], see Fig 1. These two steering modes, termed on- and off-response, are characterized by low and high values of the the rate *γ* of helix bending in the direction of the local concentration gradient, respectively, see Fig 1C. Cells were observed to employ on-responses when their helix axis pointed in the direction of the concentration gradient, but initiated a transient off-response if their helix axis pointed down the gradient [16], see Fig 1D. Here, we defined the start of an off-response with ‘high-gain’ steering as the level crossing of *γ* above its median, and recorded the angle Ψ between the tangent of the helix centreline and the local concentration gradient at the respective times, see S1 Appendix in Supporting Information for details. Note that the relationship between bending rate *γ* and orientation angle Ψ cannot be explained by the simple geometric relation *γ* ∼ sin Ψ predicted by a previous theory [15], see Fig A in S1 Appendix.

For simplicity, we will employ an idealized description with two distinct steering modes, each characterized by a constant value of a sensori-motor gain factor introduced below, in contrast to a continuous regulation of this variable.

Our theory provides a strong rationale that dynamic switching between steering modes increases the probability to find the egg in the presence of noise.

### Theory of helical chemotaxis

We consider a theoretical description of sperm chemotaxis along helical paths, which describes the feedback loop between swimming, chemotactic signalling, and steering [15, 26]. We extend this theory by incorporating a situation-specific modulation of the sensori-motor gain factor, which can take two different values in our theory. The sensori-motor gain factor, *ρ*, controls the strength of chemotactic steering in response to noisy gradient measurements by coupling the output of the chemotactic signalling system to swimming behaviour. Switching between two values of *ρ* represents a simple implementation of decision making.

**Fig 2:**
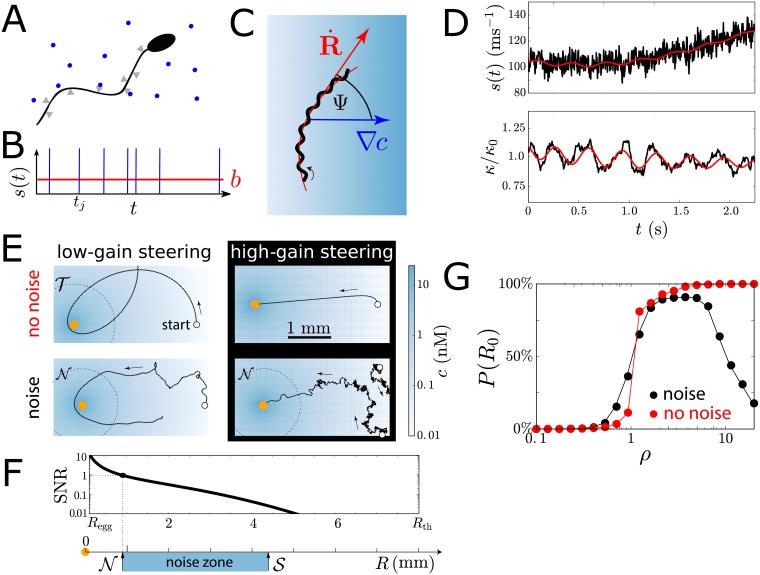
Helical chemotaxis in the presence of sensing noise. (A) Chemoattractant molecules bind to receptors on the cell membrane. (B) The sequence of binding events defines a stochastic input signal *s*(*t*) with rate *b*(*t*), Eq 1. (C) A sperm cell swims along a helical swimming path (black), whose centreline (red) can bend in the direction of a concentration gradient (blue). (D) Helical swimming in a concentration gradient causes a periodic modulation of the rate *b*(*t*) of binding events (red). Representative realization of input signal *s*(*t*) (black, low-pass filtered for visualization). This signal dynamically regulates the path curvature *κ*(*t*), here shown in the absence of sensing noise (red) and for stochastic input signal (black). (E) Example swimming paths with and without sensing noise for two values of the gain factor (‘low-gain’ steering *ρ*_low_ = 1, ‘high-gain steering’ *ρ*_high_ = 10). Egg cell (yellow disk). (F) Signal-to-noise ratio (SNR) as a function of distance *R* from the egg. The SNR defines a ‘noise zone’ spanning intermediate distances *R*, bounded by a noise zone boundary N, where SNR = 1, and a spatial limit of chemosensation S, where *c*(*R*) = (λ*T*)^−1^. (G) Probability to find the egg as a function of gain factor *ρ* for initial distance *R*_0_ = 3 mm to the egg (and random initial orientation). Without sensing noise, the success probability increases monotonically with *ρ*, while in the presence of noise, this probability displays a maximum at an optimal *ρ*. Maximum search time 300s. Error bars smaller than symbols. Parameters chosen to match experiment, see S1 Appendix.

During chemotactic navigation, a sperm cell measures the concentration of chemoattractant along its swimming path **r**(*t*). At low concentrations, the rate *b*(*t*) at which chemoattractant molecules bind to receptors on the cellular membrane is proportional to the local concentration *c*(**r**(*t*)), i.e.

b(t)=λc(r(t)) (1)

with binding constant λ = 7 s^−1^ pM^−1^ [27]. The input *s*(*t*) to the chemotactic signalling system is given by the train of individual binding events with rate *b*(*t*) (which represents an inhomogeneous Poisson process with arrival times *t*_*j*_)

$$s\left(t\right)=\sum_{j}\delta \left(t-t_{j}\right),\;\;\;\left\langle s\left(t\right)\right\rangle =b\left(t\right),$$ (2)

see Fig 2A and 2B. We employ a minimal description of chemotactic signalling with a dimensionless output of the signalling system *a*(*t*) and a dynamic sensitivity *p*(*t*) [26], which implements its main characteristics observed in experiments: sensory adaptation and relaxation to a rest state after transient stimulation [28]

$$\begin{array}{ccc} \mu \;\dot{a} & = & p[\lambda c_{b}+s\left(t\right)]-a,\\ \mu \;\dot{p} & = & p(1-a). \end{array}$$ (3)

Here, *c*_*b*_ sets a threshold of sensory adaption and *μ* characterizes a time scale of relaxation and adaptation. Dots denote time derivatives. For oscillatory input, *s*(*t*) = *s*_0_ + *s*_1_ cos(Ω*t*), the output *a*(*t*) oscillates around its steady-state value 1 with amplitude proportional to *s*_1_/(λ*c*_*b*_+ *s*_0_).

For chemotactic steering, the output of the signalling system, *a*(*t*), dynamically regulates the curvature *κ*(*t*) and torsion *τ*(*t*) of the helical swimming path

$$\begin{array}{ccc} \kappa (t) & = & \kappa_{0}-\rho \;\kappa_{0}\left(a-1\right),\\ \tau (t) & = & \tau_{0}+\rho \;\tau_{0}\left(a-1\right). \end{array}$$ (4)

Curvature and torsion uniquely characterize the time evolution of the swimming path **r**(*t*) by the Frenet-Serret equations, see S1 Appendix. For constant path curvature and torsion, *κ*(*t*) = *κ*_0_ and *τ*(*t*) = *τ*_0_, the swimming path would be a perfect helix with radius $r_{0}=\kappa_{0}/\left(\kappa_{0}^{2}+\tau_{0}^{2}\right)$, pitch $2\pi h_{0}=2\pi \tau_{0}/\left(\kappa_{0}^{2}+\tau_{0}^{2}\right)$, and angular helix frequency $\Omega_{0}=v{\left[\kappa_{0}^{2}+\tau_{0}^{2}\right]}^{1/2}$, where *v* denotes a constant swimming speed. In a concentration field, both *κ* and *τ* are dynamically regulated in response to the stochastic input signal *s*(*t*). The sensori-motor gain factor *ρ* in Eq 4 sets both the speed of chemotactic steering and of noise amplification, and will be at our focus in the following.

The chemotaxis paradigm embodied in Eqs 1–4 is summarised in Fig 2C and 2D: Helical swimming around a centreline **R** with helix axis perpendicular to a concentration gradient ∇*c* results in oscillations of the binding rate *b*(*t*) with the frequency Ω_0_ of helical swimming. As a consequence, path curvature and torsion oscillate, causing the helix to bend in the direction of the gradient. This decreases the angle Ψ between the helix axis and the gradient direction. Molecular shot noise in concentration measurements adds stochasticity to this directed steering, as discussed next.

### High-gain steering amplifies sensing noise

Eqs 1–4 (with Eqs. S4-S6 in S1 Appendix) represent a closed control loop and can be simulated numerically to obtain sperm swimming paths. We use a representative concentration field *c*(**x**), established by diffusion from a spherical source representing an egg. Parameters have been chosen to match experiment with swimming speed *v* = 200 *μ*m/*s*, helix radius *r*_0_ = 7.5 *μ*m, helix pitch 2*πh*_0_ = 48.3 *μ*m, helix period *T* = 0.34s [16], and egg radius *R*_egg_ = 100 *μ*m [29], see S1 Appendix. We use measured values for the chemoattractant content of egg cells and the diffusion coefficient of the chemoattractant [28]. Thus, computed concentrations and corresponding noise levels are representative of physiological conditions in sea urchin.

Fig 2E shows swimming paths both in the absence and presence of sensing noise, for a low and a high value of the gain factor *ρ* in Eq 4, respectively. For ‘low-gain’ steering, and in the absence of noise, i.e. *s*(*t*) = *b*(*t*), the model sperm cell initially moves closer to the egg (although it eventually misses the egg). If the helix axis is initially perpendicular to the gradient direction, the same occurs for all initial conditions with egg distance *R*_0_ = |**R**(*t* = 0)| in a range $T<R_{0}<A_{\text{low}}$, with T≈1.0mm and $A_{\text{low}}\approx 3.8\;\text{mm}$, see Figure C in S1 Appendix. For initial distances outside this attraction zone, $R_{0}>A_{\text{low}}$, swimming paths move away from the egg due to insufficient chemotactic attraction. In a ‘target zone’ defined by $R_{0}<T$, the direction of the concentration gradient changes on short length scales due to the radial symmetry of the concentration field, and helix bending during ‘low-gain’ steering is too slow to follow the gradient.

In the presence of noise, swimming paths become stochastic. For ‘low-gain’ steering, with only slight course correction, noise in the input signal hardly affects swimming paths. In contrast, a high gain factor results in fast bending of helical paths, yet it amplifies noise in concentration measurements considerably. This is particularly evident in a ‘noise zone’ spanning intermediate distances *R* from the egg, where concentration signals are detectable, but the signal-to-noise ratio (SNR) of gradient measurements is below one, see Fig 2F. We define the SNR as the ratio between the power of the gradient signal (here encoded in oscillations of the binding rate *b*(*t*) with amplitude λ|∇*c*|*r*_0_ for swimming perpendicular to the gradient direction), and the noise strength of the input signal *s*(*t*) relative to a single helix period of duration *T*

$$\text{SNR}\left(R\right)=\frac{\left(\lambda |\nabla c\right|r_{0}{)}^{2}/2}{\lambda c_{0}/T},$$ (5)

see S1 Appendix. We introduce the distance N where the SNR equals one. Additionally, we introduce a distance S where only one molecule will be detected per helical turn on average, which marks a spatial limit of chemosensation. These two distances provide a formal definition of the ‘noise zone’ as the range of distances N<R<S bounded by N and S.

### Optimal chemotactic gain factor

We computed the probability *P*(*R*_0_) to find the egg for a given initial distance *R*_0_ from the egg for a static gain factor *ρ* in Eq 4, see Fig 2G (assuming an isotropic distribution of initial swimming directions). In the hypothetical case of noise-free concentration measurements, the success probability is a monotonically increasing function of *ρ*. For physiological levels of sensing noise, however, we predict an optimal value of the gain factor *ρ* that maximizes *P*(*R*_0_), reflecting the competition between responding accurately (low *ρ*) or responding fast (high *ρ*).

### Speed of steering and directional fluctuations are inseparably coupled

The centreline **R** of helical paths describes a stochastic trajectory with directional persistence. The dynamics of its tangent vector $\dot{R}/\left|\dot{R}\right|$ can be formally described as a superposition of (i) bending in the direction of the local concentration gradient with bending rate *γ*, and (ii) effective rotational diffusion with rotational diffusion coefficient *D*, see S1 Appendix. The bending rate *γ* characterizes a noise-averaged steering response, corresponding to the expectation value *b*(*t*) of the input signal *s*(*t*), whereas the rotational diffusion coefficient *D* characterizes directional fluctuations of the tangent vector that arise from fluctuations of the input signal around its expectation value. An analytical theory valid in the limit of weak concentration gradients with |∇*cr*_0_|/*c* ≪ 1 provides expressions for both *γ* and *D*, demonstrating how both quantities scale with the sensori-motor gain factor *ρ*

$$\gamma =\frac{\rho \varepsilon }{T}\;\frac{|\nabla_{⊥}c|r_{0}}{c_{b}+c},$$ (6)

$$D=(\frac{\rho \varepsilon }{T}{)}^{2}\;\frac{c}{\lambda {\left(c_{b}+c\right)}^{2}}.$$ (7)

Here, $\varepsilon =2\pi \kappa_{0}\tau_{0}/\left(\kappa_{0}^{2}+\tau_{0}^{2}\right)$ is a geometric factor characterizing helical swimming. Eqs 6 and 7 were previously derived for the special case of a linear concentration field [15, 26] and generalized here to arbitrary concentration fields. Note that the effective rotational diffusion coefficient *D* depends on the concentration *c* of signalling molecules.

The ratio between the bending rate *γ* and the effective rotational diffusion coefficient *D* is directly related to the signal-to-noise ratio SNR defined in Eq 5

$$\frac{\gamma^{2}}{D}=\frac{2{\text{sin}}^{2}\Psi }{T}\cdot \text{SNR}.$$ (8)

Eq 8 implies that the speed of steering (characterized by *γ*) and the strength of directional fluctuations due to sensory noise (characterized by *D*) are inseparably coupled.

### Chemotaxis as a decision problem

Prompted by recent experiments [16] displayed in Fig 1, we now address dynamic switching between modes of ‘low-gain’ and ‘high-gain’ steering. We consider sperm navigation as a decision problem, in which a single chemotactic agent, here the sperm cell, can choose between two actions, i.e. ‘low-gain’ or ‘high-gain’ steering, at each state. We ask for a strategy that maximizes the probability to find the egg. We discretize phase space and map the stochastic dynamics of sperm chemotaxis on a finite-state Markov decision process (MDP) [30].

**Fig 3:**
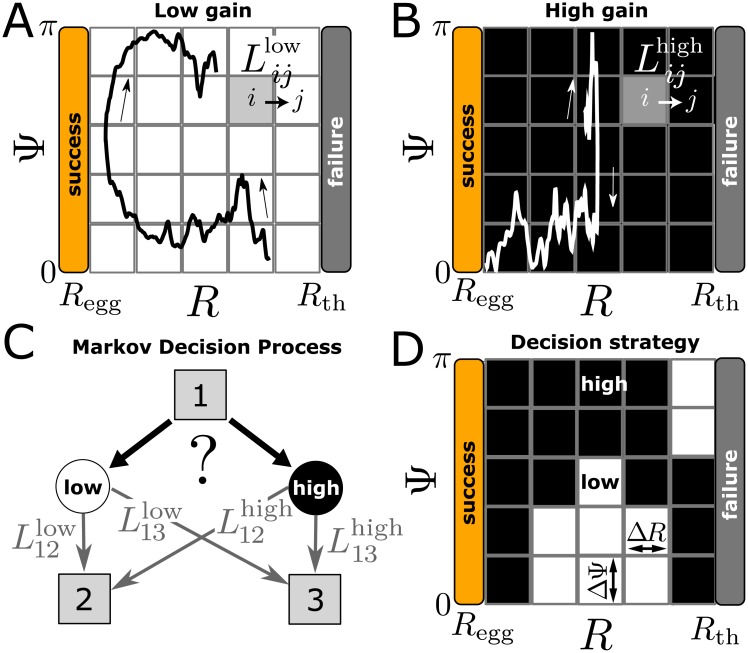
Sperm navigation mapped on a Markov decision process. (A,B) Binning of (*R*, Ψ)-phase space and sketch of trajectories for ‘low-gain’ (white) and ‘high-gain’ (black) steering. (C) Illustration of a single decision: Starting in a state 1, the player first chooses between two actions, i.e. ‘low-gain’ steering or ‘high-gain’ steering. This choice determines the transition probabilities *L*_*ij*_ for jumping to a different state, here labelled 2 and 3. (D) Illustration of a memoryless decision strategy, assigning a choice of action to each state. The figure shows coarse bins for sake of illustration.

A coarse-grained, analytical theory of sperm chemotaxis implies that the distance to the egg *R*(*t*) = |**R**(*t*)|, and the swimming direction angle Ψ(*t*), see Fig 2C, are sufficient to describe the dynamics of helical chemotaxis in a radial concentration field due to symmetry [15]. We simulated 10^4^ helical swimming paths, determined *R*(*t*) and Ψ(*t*), and then computed transition probabilities in a discretized (*R*, Ψ)-phase space for two values of a constant gain factor, *ρ* = *ρ*_low_ and *ρ* = *ρ*_high_, see Fig 3A and 3B. We obtain respective transition matrices $L_{ij}^{\text{low}}$ and $L_{ij}^{\text{high}}$ for transitions from one bin labelled *i* to another bin labelled *j*, see also Fig D in S1 Appendix. In each case, the transition dynamics is approximately Markovian, see Fig E in S1 Appendix. We additionally introduce an absorbing ‘success state’ if the egg is found, and an absorbing ‘failure state’ if the cell moves beyond a threshold distance, *R*_th_, marking the end of a single search attempt. This Markov chain allows to efficiently determine the probabilities to eventually find the egg. As a control, we compare success probabilities computed using full simulations and predictions from this Markov chain, see Fig F in S1 Appendix.

Now, we introduce a MDP, where the model cell can choose in each state between the two actions ‘low-gain’ steering and ‘high-gain’ steering, see Fig 3C. The choice determines the transition probabilities to the next state. We ask for the optimal decision strategy that maximizes the probability to eventually reach the ‘success state’. An example strategy is sketched in Fig 3D, assigning a choice of steering mode to each state. A fundamental theorem in the theory of MDP states that the optimal strategy can always be chosen to be memoryless, with a hard-wired choice for each state, independent of the history of previous states [31]. We now compute optimal memory-less strategies, and discuss how these depend on the presence of sensing noise.

### Decision making increases success probability

We computed optimal decision strategies for the MDP of sperm navigation, see S1 Appendix for details. We used the open-source probabilistic model checking software PRISM, which offers efficient algorithms to compute optimal strategies even for large MDPs [32].

**Fig 4:**
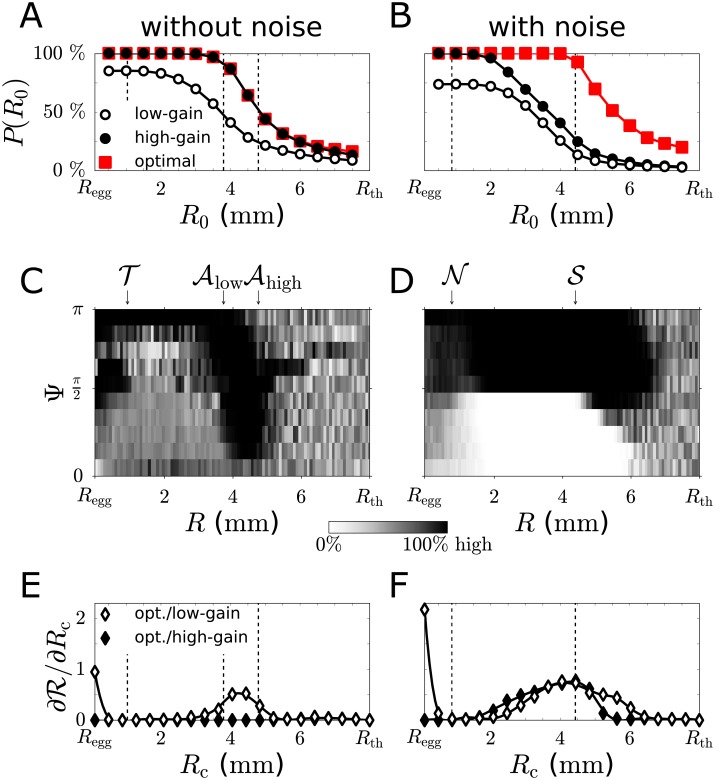
Chemotactic success with decision making. Success probability *P*(*R*_0_) for the optimal decision strategy, resulting from switching between ‘low-gain’ and ‘high-gain’ steering, as function of initial distance *R*_0_ to the egg for the case of noise-free concentration measurements (A), and physiological levels of sensing noise (B) (red squares). For comparison, success probabilities for strategies without decision making are shown (circles). (C,D) Optimal decision strategies for the cases shown in panel A and B. Greyscale represents prediction frequency of ‘high-gain’ steering, using a cohort of MDPs obtained by bootstrapping, see S1 Appendix for details. Arrows and dashed lines indicate zone boundaries as introduced in Fig 2. (E,F) Spatial sensitivity analysis of optimal strategies: Shown is the change in chemotactic range R as function of cut-off distance *R*_*c*_ for hybrid strategies that employ the optimal strategy for *R* < *R*_*c*_, and either ‘low-gain’ steering (white circles) or ‘high-gain’ steering (black circles) else. Positive values indicate a benefit of decision making at the respective distance to the egg. Parameters, see S1 Appendix.

In Fig 4, we compare the success probability for the optimal strategy to the success probabilities one would obtain for strategies that choose either always ‘low-gain’ or ‘high-gain’ steering. In the hypothetical case of noise-free concentration measurements, the performance of the optimal strategy is virtually indistinguishable to that of ‘high-gain’ steering, see Fig 4A. In contrast, when accounting for physiological levels of sensing noise, success probabilities for the optimal strategy are substantially higher than success probabilities for ‘low-gain’ and ‘high-gain’ steering, see Fig 4B. This concerns especially intermediate initial distances from the egg, where concentrations are low and sensing noise corrupts concentration measurements.

### Decision making important in noise zone

Next, we analysed significance and benefit of optimal decision making at different distances from the egg. We averaged computed strategies for an ensemble of realizations of the MDP, each with transition probabilities obtained by bootstrapping from a large cohort of simulated sperm swimming paths, see Fig 4C and 4D. Greyscale values indicate the frequency that ‘high-gain’ steering is predicted to be optimal for a given state. Thereby, we explicitly harness numerical variations in transition probabilities to extract relevant features of optimal decision strategies.

For the case of noise-free concentration measurements, we find two distinct state-space regions, where ‘high-gain’ steering is always favoured. The first region corresponds to the ‘target zone’, defined as R<T, where the model sperm cell cannot come closer to the egg if it employs ‘low-gain’ steering and initially starts with its helix axis perpendicular to the concentration gradient. A second region is bounded from below by the attraction radius $A_{\text{low}}\approx 3.8\;\text{mm}$ for ‘low-gain’ steering, and by an analogously defined $A_{\text{high}}$ for ‘high-gain’ steering with $A_{\text{high}}\approx 4.8\;\text{mm}$ from above. In this region, ‘high-gain’ steering is important to attract cells that would otherwise move away from the egg, see S1 Appendix.

In the presence of sensing noise, we consistently find that the optimal strategy chooses ‘low-gain’ steering while moving up-gradient, but chooses ‘high-gain’ steering when accidentally moving down-gradient. This choice is prevalent in the ‘noise zone’ at intermediate distances from the egg, where gradients are detectable, but the SNR ratio is below one. This theoretical prediction matches the observed steering behaviour of sperm cells in recent experiments performed at high chemoattractant concentrations [16], see Fig 1D.

To compare the efficacy of different decision strategies, we introduce the effective chemotactic volume

$$\frac{4}{3}\pi \;R^{3}=\int_{0}^{\infty }dR\;\left(4\pi R^{2}\right)P\left(R\right),$$ (9)

which defines an *effective chemotactic range* R for a given decision strategy. The chemotactic range R sets an effective target size. We find R≈6.2 mm for the optimal strategy, while $R_{\text{low}}\approx 3.8$ mm and $R_{\text{high}}\approx 4.3$ mm for a strategy that always chooses either ‘low-gain’ or ‘high-gain’ steering, respectively.

Next, we asked at which distances to the target decision making is most important. To quantify respective benefits, we computed chemotactic ranges $R(R_{c})$ as a function of a cut-off distance *R*_*c*_ for hybrid strategies. These hybrid strategies employ the optimal decision strategy only at distances smaller than *R*_*c*_, but choose always either ‘low-gain’ or ‘high-gain’ steering, respectively, outside this range. In particular, positive values of the derivative $\partial R/\partial R_{c}$ reveal at which distances decision making is most beneficial, see Fig 4E and 4F. Distances where this spatial significance measure is positive match exactly those regions where decision strategies are most stable with respect to numerical noise. Thus, two independent spatially-resolved sensitivity measures for optimal strategies give congruent results.

### Cellular implementation of decision making

While the formalism of MDPs allows us to efficiently compute optimal decision strategies, it is not evident how a biological cell would implement such strategies. In particular, a swimming cell has no direct access to the state variables *R* and Ψ, but only to the noisy concentration signal *s*(*t*). We present a minimal signalling system that implements decision making on the basis of *s*(*t*), i.e. on the basis of available information. We introduce a trigger variable *q*(*t*) that tracks the output of the signalling system *a*(*t*) with a relaxation time scale *η* [16]

$$\eta \dot{q}=a-q.$$ (10)

**Fig 5:**
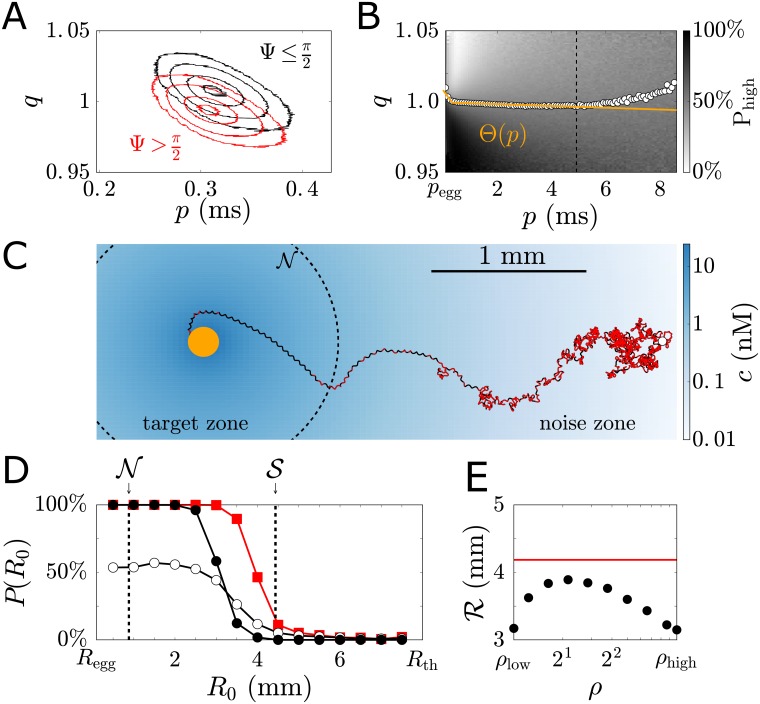
Simple implementation of optimal decision making. (A) Signalling variables *p* and *q* contain information about the helix orientation angle Ψ and distance *R* to the target. Contour levels for conditional probability densities $P(p,q|R,\Psi >\frac{\pi }{2})$ (red) and $P(p,q|R,\Psi \leq \frac{\pi }{2})$ (black) (corresponding to 1%, 10%, 50%, 90% percentiles; *R* = 1.5mm). (B) Relative frequency of ‘high-gain’ steering predicted by the optimal decision strategy, for given combination of (*p*, *q*). We define a decision boundary Θ(*p*) (yellow) by a piecewise linear fit to the 50%-contour line (up to *p* = 5ms, corresponding to a limit of sufficiently reliable state estimation, see S1 Appendix). (C) Simulated swimming path using this decision rule with dynamic switching between ‘high-gain’ steering (red) and ‘low-gain’ steering (black); projected on *xy*-plane. The chemoattractant concentration in this plane is shown (blue gradient), together with the boundary of the noise zone. (D) Success probability *P*(*R*_0_) for full simulations with simple decision making (red) as a function of initial distance *R*_0_ to the egg. For comparison, success probabilities for ‘low-gain’ steering (white) and ‘high-gain’ steering (black) are shown. (E) The effective chemotactic range R with decision making (red) is larger than R for an optimal constant gain factor (black). Parameters, see S1 Appendix.

This low-pass filter attenuates fast oscillations of *a*(*t*) caused by helical swimming in a concentration gradient, yet faithfully retains changes in the baseline of *a*(*t*), which occur for either up-gradient or down-gradient swimming (due to a finite time scale of sensory adaptation [16]). In the absence of noise, and for a given concentration field *c*(*R*), the signalling variables (*p*, *q*) are directly related to the state variables (*R*, *ψ*) as *p*^−1^ = λ[*c*_*b*_+ *c*(*R*)] and *q* = 1 + *μ*Ω_0_*h*_0_ *p*λ|*dc*(*R*)/*dR*|cos*ψ* (if we neglect residual oscillations of *q*(*t*)). In the presence of noise, *p* and *q* scatter around their expected values, see Fig 5A. Consequently, estimation of state (*R*, *ψ*) based on (*p*, *q*) is associated with an error. The accuracy in discriminating between swimming up-gradient (*ψ* ≤ *π*/2) and down-gradient (*ψ* > *π*/2) decreases as a function of distance *R* from the egg, see Fig G in S1 Appendix. Estimation of helix orientation angle Ψ can be considered feasible up to a maximal distance *R* ≈ 3mm, where the accuracy equals 66% (100%: perfect discriminability, 50%: complete lack of discriminability).

We now design a decision rule in terms of *p* and *q*

$$\rho \left(p,q\right)=\{\begin{array}{cc} \rho_{\text{low}}, & \;\text{for}\;q\geq \Theta (p)\\ \rho_{\text{high}}, & \;\text{for}\;q<\Theta (p) \end{array},$$ (11)

with decision boundary Θ(*p*) yet to be determined. From the optimal decision strategy predicted for the MDP, we compute the relative frequency of ‘high-gain’ steering for each pair of values *p* and *q*, using the likelihood of states (*R*, Ψ) for given tuple (*p*, *q*), see Fig 5B. We define Θ(*p*) as a piecewise linear fit to the 50%-contour line of this relative frequency, see S1 Appendix. Note that this decision boundary implies ‘low-gain’ steering far from the egg.

Helical sperm swimming paths simulated with this decision rule display frequent switching to ‘high-gain’ steering in the noise zone, and only sporadic events of ‘high-gain’ steering in the target zone, see representative example in Fig 5C.

Chemotaxis with decision making increases the probability of success for intermediate initial distances to the egg, similar to our analysis of the MDP, see Fig 5D. Note that we use a finite search time of 300s in Fig 5D, which yields lower success probabilities as the corresponding MDP representation, which considers infinite search times. Our simple implementation of decision making is more effective than any constant gain factor, see Fig 5E. While we compute Θ(*p*) for a specific concentration field, the same decision boundary performs superior also in other concentration fields, highlighting a general benefit of decision making, see Fig H in S1 Appendix.

## Discussion

We developed a theory of optimal chemotaxis towards a single target in the presence of noise, using sperm chemotaxis along helical paths as application example. We show that a situation-specific switching between two different steering modes—‘low-gain’ and ‘high-gain’ steering—maximizes the probability to find a target, such as an egg, at the centre of a radial concentration field of signalling molecules. The benefit of decision making is causally related to noise in sensory input. If cells could measure concentrations with perfect accuracy, decision making would provide no benefit, compared to exclusive high-gain steering.

For physiological noise levels relevant for sperm chemotaxis, ‘low-gain’ steering is chosen in the optimal strategy if the cell is approximately heading in target direction. This minimizes the risk of inadvertently steering in the wrong direction by amplifying noise in the chemotactic input signal. ‘High-gain’ steering is chosen if the net swimming direction is at least perpendicular to the target direction, and the potential benefit of fast steering outweighs the risk of wrong course corrections.

The optimal strategy predicted by our theory matches a surprising experimental observation recently made by Jikeli et al. [16], summarized in Fig 1. There, it was observed that sea urchin sperm cells switch between ‘low-gain’ and ‘high-gain’ steering depending on their net swimming direction relative to the local concentration gradient. Experiments were performed at high concentrations, corresponding to the target zone in our description. This minimized noise in the experiments. We propose that sperm cells employ decision making not only at the high concentrations tested in experiments, but also at lower, physiological concentrations. Our theory predicts that decision making is most beneficial there.

By switching between ‘low-gain’ and ‘high-gain’ steering, sperm cells dynamically adjust the persistence length of the centreline of their helical swimming paths. Thus, in addition to helical chemotaxis with gradual alignment of the centreline with the local gradient direction, these cells simultaneously perform a biased persistent random walk. This random walk can be interpreted as a time-continuous variant of ‘run-and-tumble’ chemotaxis, where the regulation of persistence length is analogous to the regulation of the duration of ‘runs’.

In conclusion, we find that noise in cellular gradient measurements poses a key constraint on chemotactic navigation. We report a fundamental relationship between the speed of chemotactic steering and the strength of directional fluctuations, which are caused by noise in the sensory input. The resultant trade-off between either reliable or fast steering applies to chemotactic motion with directional persistence in general, including chemotaxis by spatial comparison as employed e.g. by eukaryotic cells with crawling motility. We expect that other search problems can described by a Markov decision process in a similar fashion. The heuristic strategy predicted by our theory requires only minimal computational capacities of chemotactic agents and could inspire optimal control designs for artificial microswimmers.

## Supporting information

S1 Appendix Supporting table and figures. The appendix contains further details on numerical methods and data analysis. This includes a comparison of Markov chain dynamics and full simulations, as well as results for three additional concentration fields.
(PDF)
