# Supplementary material for: Decision making improves sperm chemotaxis in the presence of noise
Source: PLoS Comput Biol. 2018 Apr 19;14(4):e1006109. doi: 10.1371/journal.pcbi.1006109 (PMC5929576; doi:10.1371/journal.pcbi.1006109)
Supplement: S1 Appendix — The appendix contains further details on numerical methods and data analysis. This includes a comparison of Markov chain dynamics and full simulations, as well as results for three additional concentration fields. (PDF) [file pcbi.1006109.s001.pdf]

Supporting Information:
Decision making improves sperm chemotaxis in the presence of noise

Justus A. Kromer,[1] Steffen Märcker,[2] Steffen Lange,[1] Christel Baier,[2] and Benjamin M. Friedrich[1]
[1] *cfaed, TU Dresden, Dresden, Germany*
[2] *Faculty of Computer Science, TU Dresden, Dresden, Germany*
(Dated: April 24, 2018)

Experimental account of decision making in sperm chemotaxis

For the convenience of the reader, we presented in the main text experimental data on sea urchin sperm chemotaxis with distinct steering modes as previously published in [16], see also Fig. 1. We provide additional information on these experiments. In [16], a concentration field with cylindrical symmetry was established by uncaging a caged compound of the chemoattractant resact using spatial profiles of UV light. *A. punctulata* sea urchin sperm cells were tracked with high spatio-temporal resolution with digital inline holography, while swimming in three-dimensional space along helical paths. In a concentration field, these helical paths bent in the direction of the local concentration gradient. Fig. 1 shows a typical sperm swimming path as well as time series of the rate $\gamma(t)$ of helix bending. The rate $\gamma(t) = \gamma_1(t)$ is determined from the rotation rate of the tangent vector $\mathbf{h}(t)$ of the helix centreline $\mathbf{R}(t)$

$$\frac{d}{dt}\mathbf{h} = \gamma_1 \mathbf{g}_1 + \gamma_2 \mathbf{g}_2. \tag{S1}$$

Here, $\mathbf{g}_1 = \nabla_\perp c / |\nabla_\perp c|$ is a unit vector parallel to the component $\nabla_\perp c$ of the local concentration gradient perpendicular to the helix vector, where $\nabla_\perp c = \nabla c - \nabla_\parallel c$, $\nabla_\parallel c = (\nabla c \cdot \mathbf{h})\mathbf{h}$, $\mathbf{g}_2 = \mathbf{h} \times \mathbf{g}_1$. We observe repeated switching between phases of low and high rates of helix bending, respectively. We define the beginning of a 'high-gain' steering phase as the level crossing of the bending rate $\gamma(t)$ above its median. We find that the majority of 'high-gain' steering phases are initiated when the cell is swimming down-gradient, i.e. $\Psi > \pi/2$, see Fig. 1D. This result is robust with respect to the definition of the bending rate: an alternative definition using level crossings of a normalized bending rate $\gamma/\sin\Psi$, which accounts for a geometric relationship $\gamma \sim \sin\Psi$ predicted by previous theory [15], gives fully analogous results, see Fig. A. The data from [16] was used with permission. We refer to the original publication for further details on data acquisition and analysis.

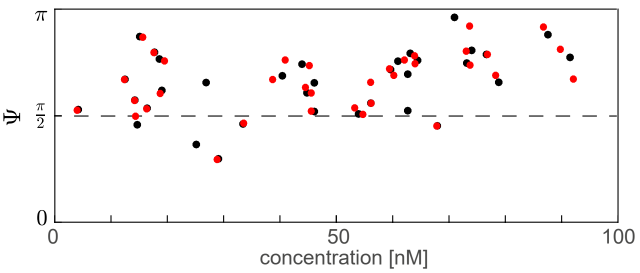

Figure A. **Decision making in chemotaxis of sea urchin sperm: supplement to Figure 1.** Scatter plot of the orientation angle Ψ and local concentration c at the beginning of 'high-gain' steering phases both for the original definition using level crossings of the bending rate γ as shown in Fig. 1D (black), and for an alternative definition using level crossings of a normalized bending rate $\gamma/\sin\Psi$ (red), ($n = 9$ cells).

Choice of parameters

We use parameters typical for helical swimming paths of *Arbacia punctulata* sea urchin sperm cells: speed $v = 200$ μm/s, mean path curvature $\kappa_0 = 0.065\,\mu\text{m}^{-1}$, and mean path torsion $\tau_0 = 0.067\,\mu\text{m}^{-1}$ [16]. The equivalent radius and pitch of helical swimming paths read $7.5\,\mu$m and $48.3\,\mu$m, respectively. The corresponding helix period is $T = 0.34$ s. Binding of chemoattractant molecules can be considered diffusion-limited with binding constant $\lambda = 7\,\text{s}^{-1}\,\text{pM}^{-1}$ [27]. The signalling time scale μ is determined as $\mu = 0.054$ s to give the optimal phase-lag of π and 0, respectively, between oscillations of path curvature and torsion with respect to oscillations of the chemoattractant signal. These phase-lags are optimal for helical chemotaxis [15] and were observed in experiments [16]. The adaptation threshold is set as $c_b = 10$ pM. At the concentration c_b, about 20 chemoattractant molecules would diffuse to a sperm cell during one helical turn. If not stated otherwise, two values of the gain factor are used, $\rho_{\text{low}} = 1$ and $\rho_{\text{high}} = 10$, which reproduce typical bending rates of helical swimming paths observed in experiments [16]. We verified that the results presented in the main text do not change qualitatively if other values for ρ_{low} and ρ_{high} are used ($\rho_{\text{low}} = 0.5$, $\rho_{\text{high}} = 5$; $\rho_{\text{low}} = 1$, $\rho_{\text{high}} = 5$). Egg cells are approximated as spheres of radius $R_{\text{egg}} = 100\,\mu$m [29] and contain approximately $n = 1.65 \cdot 10^{10}$ molecules of the chemoattractant resact [28]. The diffusion constant D of resact in sea water at temperature 16 °C was measured as 239 μm^2/s [28]. For the implementation of cellular decision making, an averaging time scale $\eta = T$ is used in Eq. 10 to attenuate fast oscillations with period T in the variable $a(t)$.

Calculation of concentration field

To compute exemplary chemoattractant concentration fields in the vicinity of an egg cell, we solve the diffusion equation for the resact concentration $c(\mathbf{x}, t)$

$$\frac{\partial}{\partial t} c = D \Delta c. \tag{S2}$$

Boundary conditions for the integration of Eq. S2 are set by an outward flux on the surface of a spherical egg with release rate $Q(t)$. In the main text, we consider the idealized case of a constant release rate $Q = n/T_{\mathrm{release}}$ with a release time of $T_{\mathrm{release}} = 60\,\mathrm{min}$, denoted as concentration field I in the following. The corresponding radial concentration profile $c(R, t)$ was obtained by integrating Eq. S2 in spherical coordinates for a time period $T_{\mathrm{int}} = T_{\mathrm{release}}$, using an explicit Euler scheme with integration time step of 1 ms and spatial resolution 1 μm. In this SI text, we also present concentration fields for a shorter release time, as well as concentration fields for instantaneous release of all resact molecules, $Q(t) = n\,\delta(t)$. The resulting concentration fields will be referred to as

- I: continuous release, $T_{\mathrm{int}} = T_{\mathrm{release}} = 60\,\mathrm{min}$,
- II: instantaneous release, $T_{\mathrm{int}} = 60\,\mathrm{min}$,
- III: continuous release, $T_{\mathrm{int}} = T_{\mathrm{release}} = 30\,\mathrm{min}$,
- IV: instantaneous release, $T_{\mathrm{int}} = 30\,\mathrm{min}$.

We anticipate that for real egg cells, the release rate $Q(t)$ decreases continuously over time, thus representing an intermediate between the two limit cases of continuous and instantaneous release. For instantaneous release of all resact molecules, the solution to Eq. S2 is well approximated by a 3-dimensional Gaussian for times $t \gg R_{\mathrm{egg}}^2/D \approx 40\,\mathrm{s}$ after chemoattractant release

$$c_{\mathrm{IR}}(\mathbf{r}, t) = \frac{n}{(4\pi D t)^{3/2}} \exp\left(\frac{-|\mathbf{r}|^2}{4Dt}\right). \tag{S3}$$

Number densities are converted to mol/l by dividing by the Avogadro constant.

Radial concentration profiles and corresponding gradients for the four exemplary concentration fields are shown in Fig. B, panels A and B. The search time $T_{\mathrm{search}} = 5\,\mathrm{min}$ considered in the main text is substantially shorter than the integration time T_{int}; thus, concentration fields can be considered as constant in time in simulations of sperm chemotaxis to very good approximation.

Frenet-Serret equations

The swimming path $\mathbf{r}(t)$ defines a co-moving coordinate system of ortho-normal vectors consisting of the tangent $\mathbf{t}$, the normal $\mathbf{n}$, and the binormal $\mathbf{b}$. Their time evolution is described in terms of path curvature $\kappa(t)$ and torsion $\tau(t)$ by the Frenet-Serret equations, which can be conveniently expressed in matrix form as

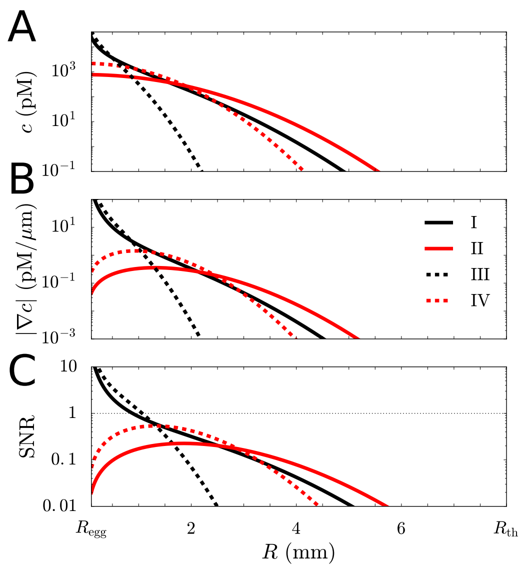

Figure B. **Exemplary concentration fields and corresponding signal-to-noise ratios.** (A) Computed concentration fields for two limit cases of chemoattractant release: continuous release with constant release rate Q (black) and instantaneous release of all n resact molecules (red) according to Eq. S3. Release times $T_{\mathbf{release}}$ are 30 min (dashed lines) and 60 min (solid lines). (B) Corresponding radial concentration gradients. (C) Signal-to-noise ratios according to Eq. 5 in the main text. Parameters: $R_{\mathrm{egg}} = 100\,\mu\mathrm{m}$ [29], $n = 1.65 \cdot 10^{10}$ molecules [28], $D = 239\,\mu\mathrm{m}^2/\mathrm{s}$ [28], $\lambda = 7\,\mathrm{s}^{-1}\,\mathrm{pM}^{-1}$ [27], $r_0 = 7.5\,\mu\mathrm{m}$ [16], $T = 0.34\,\mathrm{s}$ [16], $c_b = 10\,\mathrm{pM}$.

$$\dot{\boldsymbol{F}} = \boldsymbol{F}\boldsymbol{A}, \tag{S4}$$

with a 4×4-matrix

$$\boldsymbol{F} = \begin{pmatrix} \mathbf{t} & \mathbf{n} & \mathbf{b} & \mathbf{r} \\ 0 & 0 & 0 & 1 \end{pmatrix} \tag{S5}$$

and an infinitesimal rigid body transformation

$$\boldsymbol{A} = \begin{pmatrix} 0 & -\kappa v & 0 & v \\ \kappa v & 0 & -\tau v & 0 \\ 0 & \tau v & 0 & 0 \\ 0 & 0 & 0 & 0 \end{pmatrix}, \tag{S6}$$

where v denotes a constant swimming speed.

Simulations of stochastic swimming paths

Eqs. 1-4 and Eq. S4 were numerically integrated using an explicit Euler scheme with fixed time step $\Delta t = 10^{-3}\,\mathrm{s}$. The number n_i of chemoattractant molecules detected during the time interval $[t_i, t_{i+1}]$ is computed as a Poisson-distributed random number with expectation value $\lambda \Delta t\, c_i$, where $c_i = c(\mathbf{r}(t_i))$ denotes the local concentration at time $t_i = i\Delta t$. The spike train $s(t)$ is approximated as the constant $n_i/\Delta t$ in the interval $[t_i, t_{i+1}]$.

For numerical accuracy, the Frenet frame is propagated in time as $\boldsymbol{F}(t_{i+1}) = \boldsymbol{F}(t_i)\exp[\Delta t \boldsymbol{A}(t_i)]$, using the matrix exponential of its time propagator $\boldsymbol{A}$. This formula is exact to arbitrary order in Δt, if $\boldsymbol{A}$ can be assumed constant in the interval $[t_i, t_{i+1}]$. Numerical evaluation of the matrix exponential was done using Padé approximation. We verified that using smaller time steps did not change the statistics of results.

Time integration was performed until either the egg was found, a threshold $R_{\mathrm{th}} = 8\,\mathrm{mm}$ of maximal distance from the egg was reached, or a maximum search time of $T_{\mathrm{search}} = 5\,\mathrm{min}$ elapsed.

Signal-to-noise ratio

We quantify the impact of noise on cellular concentration measurements by a signal-to-noise ratio of helical chemotaxis (SNR), see also Eq. 5 in the main text. For a helical path with helix axis perpendicular to the concentration gradient, the stochastic chemotactic signal $s(t)$ can be approximated as

$$s(t) \approx \lambda c_0 + \lambda |\nabla c| r_0 \cos(\Omega_0 t) + \sqrt{\lambda c_0}\,\xi(t). \qquad \text{(S7)}$$

Here, $c_0 = c(\mathbf{R})$ denotes the concentration at the current centreline position $\mathbf{R}$ and $\xi(t)$ denotes white Gaussian noise with $\langle \xi(t) \rangle = 0$ and $\langle \xi(t)\xi(t') \rangle = \delta(t-t')$. We employed a diffusion approximation for the noise term, valid at sufficiently high concentrations.

Information about the gradient direction is encoded in oscillations of $\langle s(t) \rangle$. The power of these oscillations reads $(\lambda|\nabla c| r_0)^2/2$, whereas the power of the noise term in Eq. S7 with respect to a single helix period of duration T equals $\lambda c_0 / T$. Their quotient defines a signal-to-noise ratio as given in Eq. 5 in the main text. Signal-to-noise ratios for different concentration fields are shown as functions of distance $R = |\mathbf{R}|$ from the egg in Fig. B, panel C.

We introduce the distance $\mathcal{N}$ for which $\mathrm{SNR}(\mathcal{N}) = 1$, see also Table A. Broadly, this distance marks the boundary between a region close to the egg with approximately noise-free concentration measurements that enable deterministic steering, and an outer region where concentration measurements are severely corrupted by molecular shot noise.

For chemoattractant concentrations that are even lower, binding events of chemoattractant molecules become very rare, marking a limit of chemosensation. We define the distance $\mathcal{S}$ at which the concentration field $c(\mathbf{x})$ has dropped to a value $1/(\lambda T)$. At this value, only a single molecule binds per helical turn on average.

Classification of swimming paths in the absence of noise

In the absence of noise, we can distinguish three classes of simulated sperm swimming paths, depending on their initial distance $R_0 = R(t=0)$ to the egg. A model sperm cell starting with initial swimming direction perpendicular to the gradient direction i.e. $\Psi(t=0) = \pi/2$, will either

(a) for $R_0 < \mathcal{T}$: initially move away from the egg resulting in a epicycloid-like path,

(b) for $R_0 = \mathcal{T}$: orbit around the egg at constant distance,

(c) for $\mathcal{T} < R_0 < \mathcal{A}$: initially move closer to the egg, or

(d) for $R_0 > \mathcal{A}$: move away from the egg due to insufficient chemotactic attraction.

Accordingly, the minimal distance from the egg $R_{\min}$ and the maximal distance $R_{\max}$ of respective swimming paths

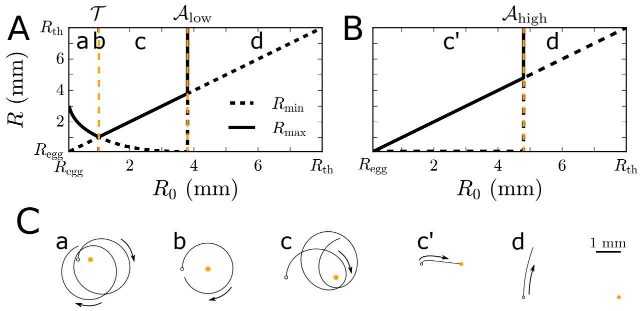

Figure C. **Classification of swimming paths in the absence of noise.** Minimal distance $R_{\min}$ and maximal distance $R_{\max}$ to the egg for simulated sperm swimming paths with initial distance R_0 and initial helix orientation perpendicular to the gradient direction with $\Psi(t=0) = \pi/2$, shown for 'low-gain' steering (A) and 'high-gain' steering (B), respectively. This representation allows us to distinguish between qualitatively different swimming paths, which are listed in panel C. Lower case letters indicate corresponding classes of swimming paths. Vertical lines in panels A and B separate regions of qualitatively different swimming paths and define critical distances $\mathcal{T} = \mathcal{T}_{\mathrm{low}}$, $\mathcal{A}_{\mathrm{low}}$, and $\mathcal{A}_{\mathrm{high}}$. White dots mark initial positions of swimming paths in panel C. We consider the case of noise-free concentration measurements with $s(t) = b(t)$, and concentration field I. Parameters: $v = 200$ μm/s, $\kappa_0 = 0.065\,\mu\mathrm{m}^{-1}$, $\tau_0 = 0.067\,\mu\mathrm{m}^{-1}$ [16], $\mu = 0.054\,$s, [15,16], $c_b = 10\,\mathrm{pM}$, $\rho_{\mathrm{low}} = 1$, $\rho_{\mathrm{high}} = 10$.

Table A. Zone boundaries for different concentration fields

concentration field	$\mathcal{N}$	$\mathcal{S}$	$\mathcal{T}_{\mathrm{low}}$	$\mathcal{T}_{\mathrm{high}}$	$\mathcal{A}_{\mathrm{low}}$	$\mathcal{A}_{\mathrm{high}}$
I	0.9	4.4	1.0	-	3.8	4.8
II	-	5.1	1.9	0.6	4.5	5.5
III	1.1	2.0	0.7	-	3.0	3.6
IV	-	3.8	1.3	0.4	3.5	4.1

All distances in units of mm. In concentration fields II and IV, sperm swimming paths escape to infinity if the initial distance R_0 is below 0.6 mm or 0.3 mm, respectively, and the initial swimming direction is perpendicular to the gradient. Definition of concentration fields I-IV, see text.

are either both equal to R_0 in case (b), satisfy $R_{\min} < R_0$ and $R_{\max} = R_0$ in case (c), and $R_{\min} = R_0$ and $R_{\max} = \infty$ in case (d), see Fig. C. In case (a), the centreline will follow an epicycloid-like path, whereas in case (c), it will follow an approximate hyperbolica.

The critical distances $\mathcal{T}$ and $\mathcal{A}$ depend on the value of the gain factor ρ. Table A displays $\mathcal{T}_{\text{low}}$, $\mathcal{T}_{\text{high}}$, $\mathcal{A}_{\text{low}}$, and $\mathcal{A}_{\text{high}}$ for $\rho = \rho_{\text{low}}$ and $\rho = \rho_{\text{high}}$ for the four concentration fields I, II, II, and IV considered in Fig. B.

Helical chemotaxis as a biased persistent random walks

We can re-state the specific example of sperm chemotaxis as a biased persistent random walk. We consider an agent moving in three-dimensional space along a path $\mathbf{R}(t)$ with constant speed v and direction $\mathbf{h}(t)$, while searching the maximum of a concentration field $c(\mathbf{x})$

$$\dot{\mathbf{R}} = \overline{v}\,\mathbf{h}, \tag{S8}$$

$$\dot{\mathbf{h}} = \gamma\,\mathbf{g}_1 + \xi_1\mathbf{g}_1 + \xi_2\mathbf{g}_2. \tag{S9}$$

Here, $\mathbf{h}$, $\mathbf{g}_1$, $\mathbf{g}_2$ represent a co-moving frame with unit vector $\mathbf{g}_1 = \nabla_\perp c/|\nabla_\perp c|$ pointing in the direction of the perpendicular component of the local concentration gradient, $\nabla_\perp c = \nabla c - (\mathbf{h}\cdot\nabla c)\mathbf{h}$, while $\mathbf{g}_2 = \mathbf{g}_1 \times \mathbf{h}$. Further, $\gamma(t)$ denotes a bending rate and $\xi_1(t)$ and $\xi_2(t)$ noise terms satisfying $\langle \xi_i(t)\xi_j(t')\rangle = 2D\,\delta_{ij}\delta(t-t')$ with noise strength D. We use Stratonovich interpretation throughout.

The case $\gamma = 0$ without steering corresponds to a persistent random walk with rotational diffusion coefficient D and persistence length $\overline{v}/(2D)$ [24]. For a chemotactic agent, we have $\gamma > 0$, resulting in a biased persistent random walk.

Previous theory shows that helical chemotaxis can be mapped on Eq. (S8) in the limit of weak concentration gradients and weak noise with γ and D given by Eqs. 6 and 7, respectively [15,16,26]. The net swimming speed $\overline{v}$ is given by $\overline{v} = v\,\tau_0/[\kappa_0^2 + \tau_0^2]^{1/2}$. Note that we consider a slightly different signalling system and restrict to the case $2\pi\mu = T$, for which no bending in $\mathbf{g}_2$ direction occurs. We further neglected a small term $\dot{\mathbf{R}}\cdot\mathbf{g}_1 + \dot{\mathbf{R}}\cdot\mathbf{g}_2$ in Eq. (S8) originally considered in [15], which has only a small effect on chemotactic navigation.

Mapping to Markov chain

We approximate chemotactic navigation with static gain factor by a discrete time *Markov chain* (MC), i.e., a stochastic process consisting of a finite set of states and transition probabilities to move from one state to another. Accordingly, the system's evolution does only depend on the current state, but not on history, i.e., the states taken previously along the system's trajectory. This property of memoryless dynamics is known as Markov property. Formally, an MC is a tuple $M = (S, \boldsymbol{L})$ where

- S is a finite set of states,
- $\boldsymbol{L} : S \times S \to [0,1]$ is a matrix of transition probabilities, whose entries L_{ij} give the probability to move from state i to state j such that $L_{i.}$ is a probability distribution.

We now present details on how the MC of sperm chemotaxis with static gain factor is defined. Each helical swimming path $\mathbf{r}(t)$ defines a centreline $\mathbf{R}(t)$, whose tangent encloses an angle $\Psi(t)$ with the local gradient direction, as well as a distance $R(t) = |\mathbf{R}(t)|$ to the target. To efficiently compute Ψ, we time-averaged local tangent vectors $\mathbf{t}$ of the swimming path over one helix period T and calculated the angle between the average tangent vector and the gradient of the concentration field at the time-averaged position. We discretize the continuous dynamics in (R,Ψ)-phase space by introducing bins with dimensions $\Delta R \times \Delta\Psi$. This set of bins defines the states of a Markov chain. We extent the state space by a 'success state' and a 'failure state', both of which are absorbing states, i.e., $L_{ii} = 1$. The transition probabilities L_{ij} from one state labelled i to another state labelled j are determined from a large ensemble of simulated trajectories by counting transitions between the two bins. These simulations employ a static gain factor ρ. Computed transition probabilities are shown for $\rho = \rho_{\text{low}}$ and $\rho = \rho_{\text{high}}$ in Fig. D.

Next, we verified that the reduction of the full dynamics to a discrete Markov chain represents a faithful approximation. We quantify deviations from Markovian dynamics, following the approach of Aït-Sahalia [33]. Let $P(i \to^2 j)$ denote the second-order transition probability, i.e., the probability to jump from state i to state j by passing through exactly one other state. For a Markov chain, this transition probability is given by the Chapman-Kolmogorov equation

$$P_{\text{Markov}}(i \to^2 j) = \sum_{k\in S} L_{ik}\,L_{kj}. \tag{S10}$$

The sum of differences between the second-order transition probability $P(i \to^2 j)$ determined from full simulations and the $P_{\text{Markov}}(i \to^2 j)$ defines a local measure for non-Markovian dynamics

$$\Delta M_i := \frac{1}{2}\sum_j \left| P(i \to^2 j) - P_{\text{Markov}}(i \to^2 j) \right|. \tag{S11}$$

For a Markov chain, we have $\Delta M_i = 0$, whereas e.g. $\Delta M_i = 1 - 1/n$ for perfectly ballistic trajectories going from state i in one out of n equally-likely directions.

Fig. E shows ΔM_i using transition probabilities determined from binned swimming paths. We find that values of ΔM_i are close to zero in almost the entire (R,Ψ)-phase space, supporting the validity of our approximation. We observe some deviations from a perfect Markov property

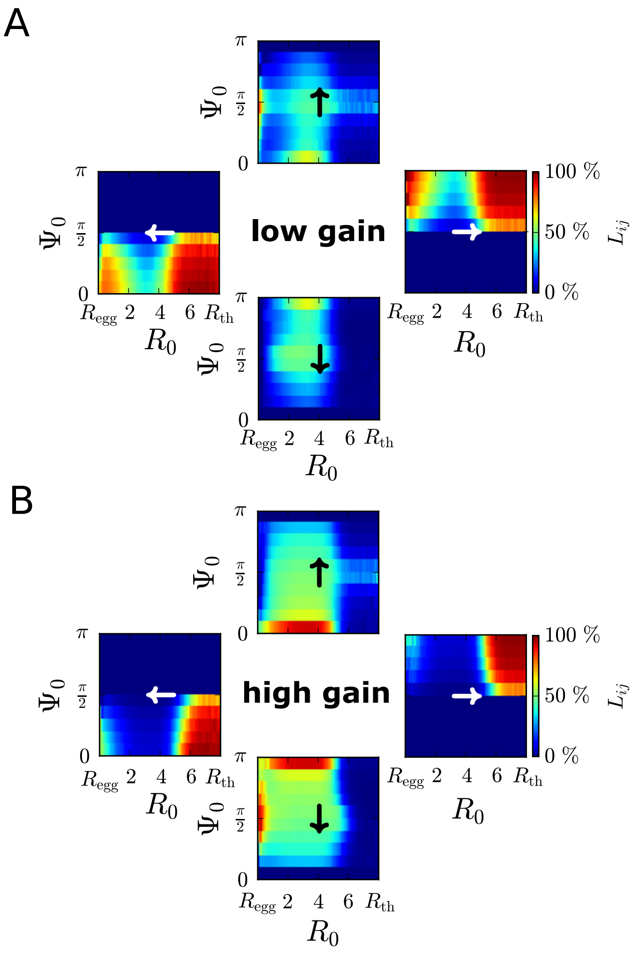

Figure D. **Transition probabilities of Markov chain.** Transitions only occur between states corresponding to neighbouring bins in (R,Ψ)-state space and can thus be visualized as density plots of probabilities for each of the four possible transition directions (indicated by arrows). Results are shown for 'low-gain' steering (A) and 'high-gain' steering (B), respectively. Transition rates were estimated from $7\cdot10^4$ binned simulated swimming paths, using gain factors $\rho=\rho_{\mathrm{low}}$ and $\rho=\rho_{\mathrm{high}}$, respectively. In both cases, concentration field I (continuous release) was used.

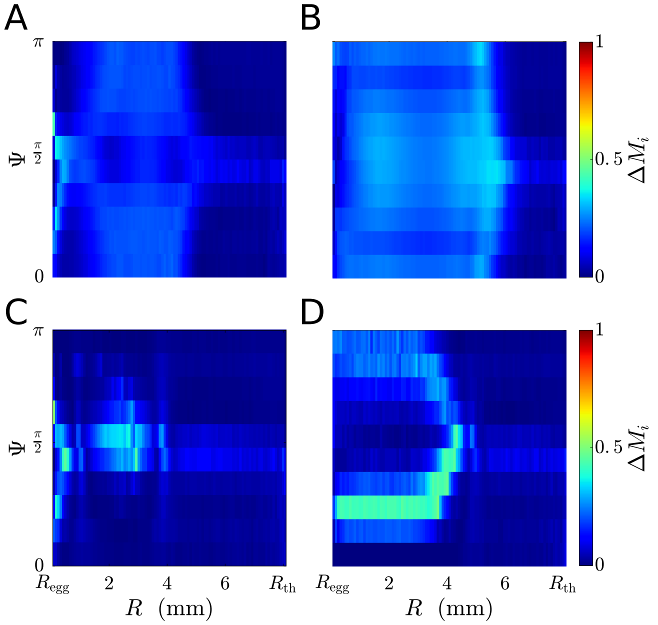

Figure E. **Deviation from Markov property.** (A,B) Local measure ΔM_i of non-Markovianity according to Eq. S11 for binned simulated sperm swimming paths with 'low-gain' steering (A) and 'high-gain' steering (B), respectively. Number of simulated swimming paths is $7\cdot10^4$. (C,D) Same as panel A and B, but for the case of noise-free concentration measurements with $s(t)=b(t)$. Number of simulated swimming paths is $6\cdot10^4$. Parameters: as in Fig. C; $\lambda=7\,\mathrm{s}^{-1}\,\mathrm{pM}^{-1}$ [27].

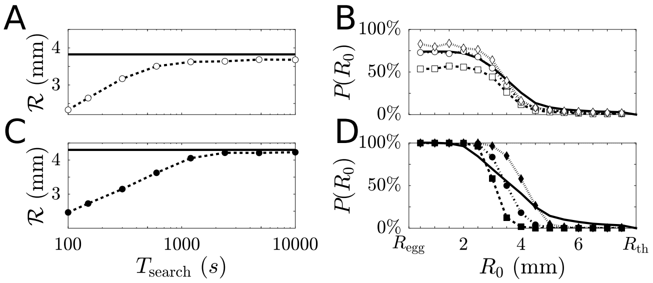

Figure F. **Comparison of Markov chain and full simulations of helical chemotaxis.** Chemotactic range $\mathcal{R}$ (panels A and C) and success probabilities $P(R_0)$ (panels B and D), computed for MC (solid lines) and from full simulations of swimming paths with different search times [dashed lines and symbols: $T_{\mathrm{search}}=300\,\mathrm{s}$ (squares), $600\,\mathrm{s}$ (circles), $10^4\,\mathrm{s}$ (diamonds)]. Panels A and B correspond to 'low-gain' steering and panels C and D to 'high-gain' steering, respectively. Parameters: as in Fig. C; $\lambda=7\,\mathrm{s}^{-1}\,\mathrm{pM}^{-1}$ [27].

in the case of 'high-gain' steering at intermediate distances from the egg. In this noise zone, single molecular detection events cause strong reorientation responses in simulated swimming paths, with corresponding ballistic motion in (R,Ψ)-phase space in Ψ-direction across several bins, resulting in slightly non-Markovian dynamics with finite memory. We find that ΔM_i values decrease if $\Delta\Psi$ is increased or if ΔR is decreased. In order to ensure both low values of ΔM_i as well as sufficient resolution and sampling of transition rates, we use a bin size of $\Delta\Psi=\pi/10$ and $\Delta R=(R_{\mathrm{th}}-R_{\mathrm{egg}})/99\approx79.8\,\mu\mathrm{m}$ throughout the manuscript.

As an additional test, we compared chemotactic ranges and success probabilities. We found that success probabilities for individual bins saturate as bin sizes ΔR and $\Delta\Psi$ decrease. In particular, saturation is reached for the bin sizes used throughout the manuscript.

Fig. F compares success probabilities for both full simulations and the corresponding MCs. For the full simulations, different search times T_{search} were employed. We find that corresponding chemotactic ranges $\mathcal{R}$ converge

to those predicted by the MC as the search time T_{search} is increased, consistent with the fact that the MC corresponds to the limit case of an infinite search time. Some differences in numerical values between MC and full simulations occur for the success probabilities $P(R_0)$ as function of initial target distance R_0. These differences result from the discretization of the dynamics using a finite number of bins as well as small deviations from perfect Markovian dynamics of the full simulations.

Formulation of Markov decision processes

A *Markov decision process* (MDP) extends a Markov chain by introducing a finite set of actions, one of which can be chosen in each time step, as well as a family of distinct transition matrices for each action. It forms a tuple $M = (S, Act, (\boldsymbol{L}^a)_{a\in Act})$, where

- Act is a finite set of *actions* (here: 'low-gain' steering or 'high-gain' steering)
- $(\boldsymbol{L}^a)_{a\in Act}$ is a *family of transition probability matrices*, indexed by the possible actions (here: L_{ij}^{low} and L_{ij}^{high}).

The transition probabilities L_{ij}^{low} and L_{ij}^{high} are determined as above from simulated swimming paths that employ a static gain factor that equals either ρ_{low} or ρ_{high}, respectively. Accordingly, we obtain a MDP for chemotactic navigation with decision making.

A *memoryless (or history-independent) deterministic strategy* for a MDP is a function $\Pi : S \to Act$ that specifies, which action to take in each state. Each strategy Π uniquely defines a MC, whose transition matrix $\boldsymbol{L}$ has entries selected from the transition matrices for the different actions according to the strategy Π

$$L_{ij} = L_{ij}^{\Pi(i)} \text{ for all } i \in S. \tag{S12}$$

The probability x_i to eventually reach one (or several) success states, starting from state i, satisfies the linear equation

$$x_i = \sum_{j\in S} L_{ij}\, x_j, \tag{S13}$$

provided all success and failure states j are absorbing with $L_{jj} = 1$. Eq. S13 for all $i \in S$ defines a system of linear equations, which may have multiple solutions. The vector $\mathbf{x}$ of desired success probabilities is the (component-wise) least solution that satisfies $x_j = 1$ for all success states j [31].

Calculation of optimal decision strategies

We employ techniques from *probabilistic model checking*, a formal method for the qualitative and quantitative analysis of stochastic systems [31] and [34]. Of note, maximal probabilities and a corresponding optimal memoryless strategy can in principle be computed exactly in polynomial time [31], e.g., by rewriting the Bellman equation S14 below into a linear program. However, approximation methods as employed here often perform considerably better in applications, especially for large models typically considered in probabilistic model checking. We used the open source probabilistic model-checker Prism [32], employing a *value iteration* algorithm [30] and [35] to approximate the maximal probability to reach the success state and to compute an optimal memoryless strategy. The *value-iteration* algorithm builds on the idea of backward induction and is initialized with a probability vector $\mathbf{x}^{(0)}$, whose components are all zero, except $x_{\text{success}}^{(0)} = 1$ for the component corresponding to the success state. Then, the vectors $\mathbf{x}^{(1)}$, $\mathbf{x}^{(2)}$, $\mathbf{x}^{(3)}$, ... are computed successively in terms of the *Bellman equation*

$$x_i^{(n+1)} = \max\left\{\sum_{j\in S} L_{ij}^a\, x_j^{(n)} \ \middle|\ a \in Act\right\}, \tag{S14}$$

until $x_i^{(n+1)} - x_i^{(n)} < \varepsilon$ for all states i and a sufficiently small threshold ε. The maximal probabilities correspond to the limit $x_i = \lim_{n\to\infty} x_i^{(n)}$. By storing the last selected action for each state, a strategy is computed alongside. However, the termination criterion may cause the value iteration to stop prematurely and yield incorrect probabilities and strategies. Therefore, we additionally used an *interval iteration* algorithm to confirm the validity of our results. Interval iteration works similar to value iteration, but approximatively frames the exact values from below and above until an ε-environment is reached or the exact solution can be deduced [36]. Interval iteration has recently been implemented in Prism (though currently without the possibility to compute optimal strategies) [37].

To account for numerical errors that result from estimating transition matrices $\boldsymbol{L}^{\text{low}}$ and $\boldsymbol{L}^{\text{high}}$ by counting transitions between bins, we calculated optimal strategies using transition matrices estimated from 100 different sets of swimming paths. Each set consists of 10^4 swimming paths. In Fig. 4C and D, we show the relative frequency that 'high-gain' steering is determined as the optimal action in a certain state for the corresponding ensemble of MDPs. To determine the 100 sets, swimming paths were drawn randomly with replacement from a pool of $6 \cdot 10^4$ (Fig. 4C) and $7 \cdot 10^4$ (Fig. 4D) swimming paths, respectively. The number of outgoing transitions for each state, used to estimate transition probabilities, varies for different states: the mean numbers of transitions are ≈ 2000 (standard deviation ≈ 1900) for 'low-gain' steering with and without noise and ≈ 11000 (17000) 'for high-gain' steering, respectively. Furthermore, we ensure a minimum number of 5 transitions for each state by drawing a new set if this condition is not fulfilled.

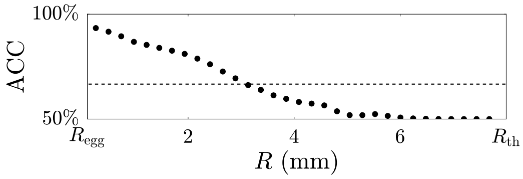

Figure G. **Accuracy of state estimation in full simulations.** Accuracy ACC for discriminating between up-gradient and down-gradient swimming, according to Eq. S16, as function of distance R from the egg. We chose a cutoff at ACC = 66% (dashed line) to mark a limit of reliable state estimation. Statistics was obtained using 10^5 simulated swimming paths with binning $\Delta p = 0.014$ ms and $\Delta q = 0.02$.

Cellular implementation of decision making: determination of decision boundary

We first determined the base probability $P(R, \Psi|p, q)$ that a cell is in state (R, Ψ) if it observes p and q. For this, we simulated a set of $8 \cdot 10^4$ swimming paths with constant gain $\rho = \rho_{\rm low}$ and simultaneously recorded R, Ψ, p, and q. The base probability was computed as a bivariate histogram using bin sizes $\Delta q = 10^{-3}$ and $\lambda \Delta p = 6 \cdot 10^{-4}\,\text{pM}^{-1}$.

We then calculated a likelihood $\mathcal{L}(p, q)$ for 'high-gain' steering as a function of (p, q), using the predicted frequency of 'high-gain' steering from the MDPs as a function of (R, Ψ) (Fig. 4D) and the base probability $P(R, \Psi|p, q)$. The resulting likelihood function is shown in Fig. 5B. We accurately determined the 50%-contour line of this likelihood function by locally fitting test functions to slices $\mathcal{L}(p = \text{const}, q)$ (3rd order polynomials for $p < 5\Delta p$, sigmoidal functions for larger values of p).

Finally, the decision boundary $\Theta(p)$ was determined by a piecewise linear fit

$$\Theta(p) = \Theta_0 + \begin{cases} -\alpha_1(p - p_0) & p \leq p_0 \\ -\alpha_2(p - p_0) & p > p_0 \end{cases} \qquad \text{(S15)}$$

of the 50%-contour line, using all values up to $p < p_{\rm c}$. Here, $p_{\rm c}$ denotes an upper limit for reliable state estimation, which is determined below. Fitting Eq. S15 resulted in $\Theta_0 = 0.997$, $p_0 = 0.333\,\text{ms}$, $\alpha_1 = 2.7 \cdot 10^{-2}\,\text{ms}^{-1}$, and $\alpha_2 = 0.064 \cdot 10^{-2}\,\text{ms}^{-1}$.

To assess the limits of state estimation based on (p, q), and compute the value p_c used above to select reliable values for the fit of Eq. S15, we determined the accuracy in discriminating between up-gradient swimming with $\Psi < \pi/2$ and down-gradient swimming with $\Psi > \pi/2$. For a given distance R to the egg, we compute base probabilities $P_{\rm up}(p, q|R) := P(p, q|R, \Psi < \pi/2)$ and $P_{\rm down}(p, q|R) := P(p, q|R, \Psi > \pi/2)$ for up-gradient and down-gradient swimming, respectively. The use of the conditional probabilities $P_{\rm up}$ and $P_{\rm down}$ ensures that both cases are treated equally, even if one of the two cases occurs more frequently in the underlying set of simulated swimming paths. We then compute the accuracy of the binary decision scheme based on the condition $P_{\rm up} \lessgtr P_{\rm down}$ as

$$\text{ACC} := \frac{T_{\rm up} + T_{\rm down}}{T_{\rm up} + T_{\rm down} + F_{\rm up} + F_{\rm down}}, \qquad \text{(S16)}$$

where $T_{\rm up}$ and $T_{\rm down}$ denote the frequency of correctly detecting up-gradient and down-gradient swimming, respectively, and $F_{\rm up}$ and $F_{\rm down}$ the respective frequencies for incorrect decisions. Specifically,

$$T_{\rm up} = \int_{P_{\rm up} > P_{\rm down}} dq dp \; P_{\rm up}, \quad T_{\rm down} = \int_{P_{\rm up} < P_{\rm down}} dq dp \; P_{\rm down}. \qquad \text{(S17)}$$

The accuracy as a function of distance R is shown in Fig. G. An accuracy equal to 100% indicates perfect discriminability, whereas a value of 50% indicates complete lack of discriminability. We chose a value of 66% to mark the limit of reliable state estimation. This limit corresponds to a distance $R_c \approx 3\,\text{mm}$ to the egg, at which the adaptation variable p takes the mean value $p_c \approx 5\,\text{ms}$.

Performance of cellular decision making in different concentration fields

We computed success probabilities as function of initial target distance R_0, using the simple implementation of cellular decision making embodied in Eqs. 10 and 11 with piecewise linear decision boundary Eq. S15, for the four different concentration fields I, II, III, and IV, see Fig. H. We note that decision making outperforms 'high-gain' steering for scenario I, II, IV, where concentrations gradients are sufficiently shallow and the signal-to-noise ratio is sufficiently low.

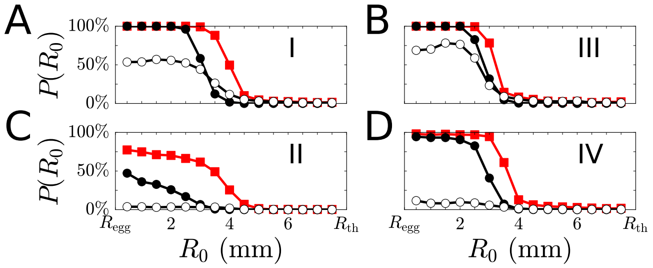

Figure H. **Performance of decision making in different concentration fields.** Computed success probabilities for cellular implementation of decision making (red squares), compared to success probabilities for static gain factor with 'low-gain' (white circles) and 'high-gain' steering (black circles), respectively. Panels A-D display results for concentration fields I – IV, respectively. The same decision strategy as in Fig. 5 is used throughout. Parameters: as in Fig. C; $\lambda = 7\,\text{s}^{-1}\,\text{pM}^{-1}$ [27], $\eta = T$.

33. Aït-Sahalia Y, Fan J, Jiang J. Nonparametric tests of the Markov hypothesis in continuous-time models. Ann Statist. 2010;38(5):3129–3163.
34. Forejt V, Kwiatkowska M, Norman G, Parker D. Automated verification techniques for probabilistic systems. In: Formal Methods for Eternal Networked Software Systems. Springer; 2011. p. 53–113.
35. Puterman ML. Markov Decision Processes: Discrete Stochastic Dynamic Programming. John Wiley & Sons; 1994.
36. Haddad S, Monmege B. Reachability in MDPs: Refining convergence of value iteration. In: International Workshop on Reachability Problems. Springer; 2014. p. 125–137.
37. Baier C, Klein J, Leuschner L, Parker D, Wunderlich S. Ensuring the Reliability of Your Model Checker: Interval Iteration for Markov Decision Processes. In: Proc. of the 29th International Conference on Computer Aided Verification (CAV). Springer; 2017. p. 160–180.
